# Beta oscillation is an indicator for two patterns of sensorimotor synchronization

**DOI:** 10.1002/pchj.696

**Published:** 2023-10-31

**Authors:** Yuelin Liu, Chen Zhao, Tillman Sander‐Thömmes, Taoxi Yang, Yan Bao

**Affiliations:** ^1^ School of Psychological and Cognitive Sciences Peking University Beijing China; ^2^ Institute of Medical Psychology Ludwig‐Maximilian‐University Munich Munich Germany; ^3^ Physikalisch‐Technische Bundesanstalt Berlin Germany; ^4^ Laboratory of Neurobiology, Division of Cell & Developmental Biology University College London London UK; ^5^ Beijing Key Laboratory of Behavior and Mental Health Peking University Beijing China

**Keywords:** beta oscillation, motor cortex, sensorimotor synchronization, time perception

## Abstract

Previous study indicates that there are two distinct behavioral patterns in the sensory‐motor synchronization task with short stimulus onset asynchrony (SOA; 2–3 s) or long SOA (beyond 4 s). However, the underlying neural indicators and mechanisms have not been elucidated. The present study applied magnetoencephalography (MEG) technology to examine the functional role of several oscillations (beta, gamma, and mu) in sensorimotor synchronization with different SOAs to identify a reliable neural indicator. During MEG recording, participants underwent a listening task without motor response, a sound‐motor synchronization task, and a motor‐only continuation task. These tasks were used to explore whether and how the activity of oscillations changes across different behavioral patterns with different tempos. Results showed that during both the listening and the synchronization task, the beta oscillation changes with the tempo. Moreover, the event‐related synchronization of beta oscillations was significantly correlated with motor timing during synchronization. In contrast, mu activity only changes with the tempo in the synchronization task, while the gamma activity remains unchanged. In summary, the current study indicates that beta oscillation could be an indicator of behavioral patterns between fast tempo and slow tempo in sensorimotor synchronization. Also, it is likely to be the potential mechanism of maintaining rhythmic continuous movements with short SOA, which is embedded within the 3 s time window.

## INTRODUCTION

Sensorimotor synchronization is the coordination of movements with an external rhythm or tempo (Bao et al., 2015; Repp & Su, 2013). Dancing or clapping to music is a typical example in daily life. Previous study shows that with a different tempo, the behavioral patterns in sensorimotor synchronization can be distinct. For example, in a typical tapping task, when the stimulus‐onset asynchrony (SOA) between the beats is less than some 2–3 s, the motor response could be predictive and usually lead beats by around 20–100 ms (e.g., Chen et al., 2002; Radil et al., 1990). However, as SOA gets longer, motor responses start lagging behind the beats (Mates et al., 1994; Miyake et al., 2007); at the same time, subjects report greater subjective difficulty (Bååth & Madison, 2012). These behavioral changes are thought to be caused by different prediction systems between short and long durations with a border in the range of 2–3 s (Miyake et al., 2007; Pöppel, 1997). However, to our knowledge, there are no concise shreds of evidence or conclusions regarding the underlying neural indicators or mechanisms of the distinct behavioral patterns. We aim in this study to explore the neural activity that signifies the transition between predictive response to reactive response in sensorimotor synchronization tasks.

On the neural level, beta activity in motor areas varies with tempo. Beta oscillations are associated with active motor inhibition (Joundi et al., 2012; Pfurtscheller et al., 2003). A decrease in the amplitude of the beta wave, event‐related desynchronization (ERD), usually occurs before and during a movement, representing the release of the motor system from inhibition. An increase in the amplitude of the beta wave, event‐related synchronization (ERS), typically follows movement termination, indicating an active return (Pollok et al., 2006). It has been observed that during sensorimotor synchronization tasks with short SOA, or fast tempo, beta oscillations remain desynchronized (Toma et al., 2002; Yuan et al., 2010). Those differences in beta activity have been interpreted as changes in movement patterns (e.g., Toma et al., 2002). During short‐SOA sensorimotor synchronization, movements are often fast and continuous. Sustained desynchronized beta oscillations are indicative of active involvement of motor areas. On the other hand, during long SOA sensorimotor synchronization, movement is discrete and needs to be initiated at each pace. In this case, beta oscillations return to baseline after each movement, which results in a larger ERD and ERS.

Recent findings indicate that beta oscillations correlate not only with different types of movement but also with the temporal structure of the task (Arnal et al., 2011; Todorovic et al., 2011). With or without overt movement, beta activity changes similarly during listening to isochronous beats (Fujioka et al., 2009; Saleh et al., 2010). Importantly, beta activity could even predict subjects' perceived rhythm and duration (Arnal & Giraud, 2012; Kononowicz & van Rijn, 2015). In line with these findings, functional magnetic resonance imaging (fMRI) studies revealed that both listening to and encoding auditory rhythms activate the motor system (Chen et al., 2008; Grahn & Brett, 2007). In light of these findings, the motor system is hypothesized to internally simulate movements synchronized with ongoing events (Patel & Iversen, 2014). The studies mentioned above indicate that the activity of beta oscillation could well be a candidate indicator to signify the behavioral pattern shift between sensorimotor synchronization tasks with short and long SOA. The difference in beta oscillation could also represent the underlying mechanism of the temporal prediction system in motor control. However, to our knowledge, this hypothesis has not been addressed before.

Besides beta oscillation, mu and gamma oscillations in the motor cortex also play a role in movement control. The power of mu oscillations declines before movements (Pfurtscheller & da Silva, 1999) contributing to active motor inhibition. Unlike beta and mu oscillations, the power of gamma oscillations increases before movements and recent research suggests they are actively involved in motor control. However, it is unclear whether mu and gamma oscillations are also sensitive to the temporal structure in sensorimotor synchronization tasks.

Taken together with the above findings, the present study aimed to examine whether beta, gamma, and mu oscillation could change along with the tempo of sensorimotor synchronization, and further explore the neural mechanism of motor control and temporal perception. Innovatively in our paradigm, we added a passive listening task before the section on sensorimotor synchronization, allowing us to observe how neural signals change with tempo even without overt movement.

## METHODS

### Participants

This study received ethics approval by the ethical board of the Ludwig Maximilian University of Munich.

Fourteen young right‐handed neurologically healthy participants took part in this study. All of them reported normal hearing, and none of the participants received professional music training. Before the experiment, all participants were given full instructions and needed to consent to participation, and they received compensation after finishing the experiment.

### Procedure

**FIGURE 1:**
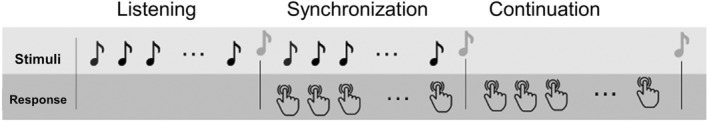
Experimental design. Participants were instructed to listen to 40 clicks and then synchronize with another 40 clicks. After that, the clicks were removed, participants kept tapping at the tempo 40 times. The gray phonic notes denote the switching clicks.

The experiment contained six blocks. In each block, participants encountered three different tasks: a listening task, a synchronization task, and a continuation task (see Figure 1). The order of tasks was fixed. First, participants listened to 40 isochronous clicks without any movement (listening task). They then synchronized to another 40 clicks with the same tempo by tapping their right index finger at click onset (synchronization task). After that, the pacing clicks were removed; participants were asked to continue tapping at the established rate for another 40 taps (continuation task). The task switch was signified by a click with a higher frequency at the end of each condition. The SOA of pacing stimuli varied by blocks (0.60, 1.20, 1.80, 3.00, and 4.20 s), representing different tempos. For each SOA, the duration of clicks was 16, 32, 48, 84, and 112 ms in proportion to the SOA. The block order was randomized for each participant. The inter‐block interval was 2 min. A 3 min resting‐state recording was conducted before the experimental session, in which participants relaxed with open eyes. Participants practiced the tasks before going into the experimental chamber. They were instructed to tap as accurately as possible and keep other body parts still.

### Data acquisition

MEG signal was recorded at a sampling rate of 500 Hz with a Yokogawa MEG system (Yokogawa Electric Corporation, Japan) situated in a dimly lit, sound‐attenuated, and magnetically shielded chamber (Ak3b, Vakuumschmelze, Hanau, Germany). It consisted of 125 axial gradiometers and three reference magnetometers. The head position with respect to the sensor helmet was measured with coils attached to the scalp at anatomical landmarks (nasion and periauricular points). The coil locations were digitized using a 3D digitizer (Zebris, Isny, Germany) based on three anatomical landmarks. Clicks (approximately 75 dB SPL) were delivered binaurally to the participants' ears via MEG‐compatible tube earphones (Etymotic Research, Elk Grove Village, USA). All stimuli were presented using Presentation software (Neurobehavioral Systems). Participants lay horizontally with arms crossed in front of their chests. They held a computer mouse in the right hand and responded by clicking a button on it.

### Data analysis

Three subjects were excluded due to extreme noise in the data.

The first three taps were discarded since stable synchronization and continuation took some time. The last tap was excluded due to possible interference from task switching.

### Behavioral analysis

First, an analysis was made to confirm whether the behavioral pattern in sensorimotor synchronization task changes with tempo. The percentage of predictive taps in the synchronization task was chosen to index the performance. To determine the predictive taps, we set a reaction time limit of 150 ms after the click onset. Predictive taps were defined as taps that fell before the reaction time limit. The independent variable was SOA (0.60, 1.20, 1.80, 3.00, and 4.20 s). Post‐hoc *t*‐tests were performed using the Bonferroni correction.

### MEG data preprocessing

MEG data were analyzed using Matlab R2018b with custom‐written scripts and the FieldTrip toolbox (Oostenveld et al., 2011; http://www.ru.nl/neuroimaging/fieldtrip/). Line noise was cleaned using a band‐stop filter (49–51, 99–101, and 149–151 Hz, Butterworth, filter order 4). Low‐frequency noises were removed by a high‐pass filter (0.5 Hz, Butterworth, filter order 4). After that, bad and excessively noisy sensors were visually inspected and interpolated from surrounding sensors (interpolated sensors: 1–3 for each subject). Eye‐related and heartbeat‐related noises were removed by independent component analysis (ICA; deleted components number: 3–7 per participant). Data were then segmented into epochs starting 2000 ms before sound or response onset and ending 2000 ms later. Finally, trials with excessive artifacts were visually detected and excluded from further analysis (deleted trials: 0–11 for each subject).

### Functional localizers for motor sensors

Since we did not have access to anatomical magnetic resonance images of individual subjects, the accuracy of the source analysis was greatly diminished (Gross et al., 2013). The first motor‐evoked field (MEF1) was chosen as a functional localizer to predefine motor‐cortex‐related areas at the topographical level. MEF1 is a typical movement‐evoked wave peaking around 50 ms after a keystroke (Joliot et al., 1998).

First, we excluded sensors near the helmet edge. Second, the trials from the continuation condition were averaged for each sensor. Then, for each participant, the five sensors with the highest MEF1 absolute peak amplitudes were selected. To calculate absolute peak amplitude, we first located the maxima between −30 and 70 ms around the tap. Then we averaged the absolute event‐related field in the time window −6 to 6 ms around maxima. This average value was taken as the peak amplitude.

### Time‐frequency analysis

The MEG time series were decomposed into time‐frequency representations of 5–80 Hz in 1 Hz steps. A 7‐cycle Morlet wavelet was used. The first and last 300 ms of data were discarded to avoid edge effects. The remaining part was then decibel normalized to minimize variation due to individual differences and the 1/*f* phenomenon. Each frequency band was then averaged over trials and motor‐cortex‐related sensors separately (mu: 10–12 Hz; beta: 14–28 Hz; gamma: 30–50 Hz).

We first examined the ERD and ERS peaks of the beta oscillation. For the ERD peak, we located the minimum in the time window between −400 and 200 ms around the tap/sound onset. The peak amplitude was calculated as the average power in the time window −6 to 6 ms around the minimum. For the ERS peak, we located the maximum in the time window from 0 to 1000 ms after the tap/sound onset. Similarly, the peak amplitude was calculated as the average power in the time window −6 to 6 ms around the maximum. Afterward, the influence of task and SOA (tempo) on the ERD and ERS peak was tested by two‐way repeated analysis of variance (ANOVA). The independent variables were task (listening, synchronization, and continuation task) and SOA (0.60, 1.20, 1.80, 3.00, and 4.20 s).

We then examined the activity in mu‐ and gamma‐frequency bands. Because it is unclear whether these two oscillations involve the processing of temporal information without overt movement, we first examined whether the power of mu and gamma oscillation changed during the listening task. Data from 500 ms before to 200 ms after the click onset were selected for each SOA condition separately. We used a *t*‐test to detect whether the mu and gamma power were significantly different from 0. The Benjamini‐Hochberg false discovery rate was used to correct the *p*‐value. Subsequently, a two‐way ANOVA was used to test for the effects of the task (synchronization and continuation tasks; or listening, synchronization, and continuation conditions if any significance was found in the listening task), SOA (0.60, 1.20, 1.80, 3.00, and 4.20 s). The dependent variables were mu ERD peak, and gamma ERS peak, respectively. The time window for the peak calculation was 500 ms before to 200 ms after click onset or movement onset. Calculations were consistent with synchronization and desynchronization peaks for the beta oscillation.

### Prediction of oscillatory activity on behavioral asynchrony

We then tested whether these neural signals could predict the performance in synchronization tasks. Using single‐trial data, we built a mixed linear regression model. Behavioral asynchrony was the dependent variable, and the independent variables were the neural activities that differed significantly across SOAs during the synchronization task. The random variable was the subject, which included a correction for the intercept. The analysis was done using the lme4 and lmerTest packages in R software (Kuznetsova et al., 2017).

## RESULTS

### The percentage of predictive taps decreased with SOA

**FIGURE 2:**
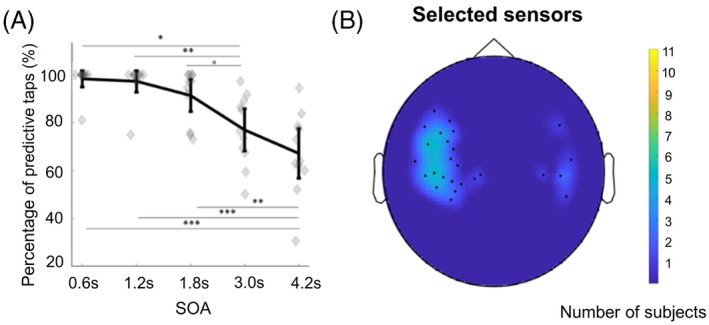
(A) Percentage of predictive taps under different SOAs. A lower percentage of predictive taps were observed when SOA was 3.0 or 4.2 s. (B) The selected sensors. The orange areas indicate that a greater number of subjects selected the sensors.

The percentage of predictive taps significantly decreased with SOA (*F*(4,40) = 26.087, *p* < .001, *η* ^2^ = 0.723; Figure 2A). Post‐hoc analysis revealed a significant difference between the long SOA (3.00 and 4.20 s) and the other short SOA (*p*3.0 vs. 0.6 = 0.015; *p*3.0 vs. 1.2 =0 .001; *p*3.0 vs. 1.8 = 0.051; *p*4.2 vs. 0.6 = 0.002; *p*4.2 vs. 1.2 < 0.001; *p*4.2 vs. 1.8 = 0.001).

### Beta activities changed more with longer SOAs

**FIGURE 3:**
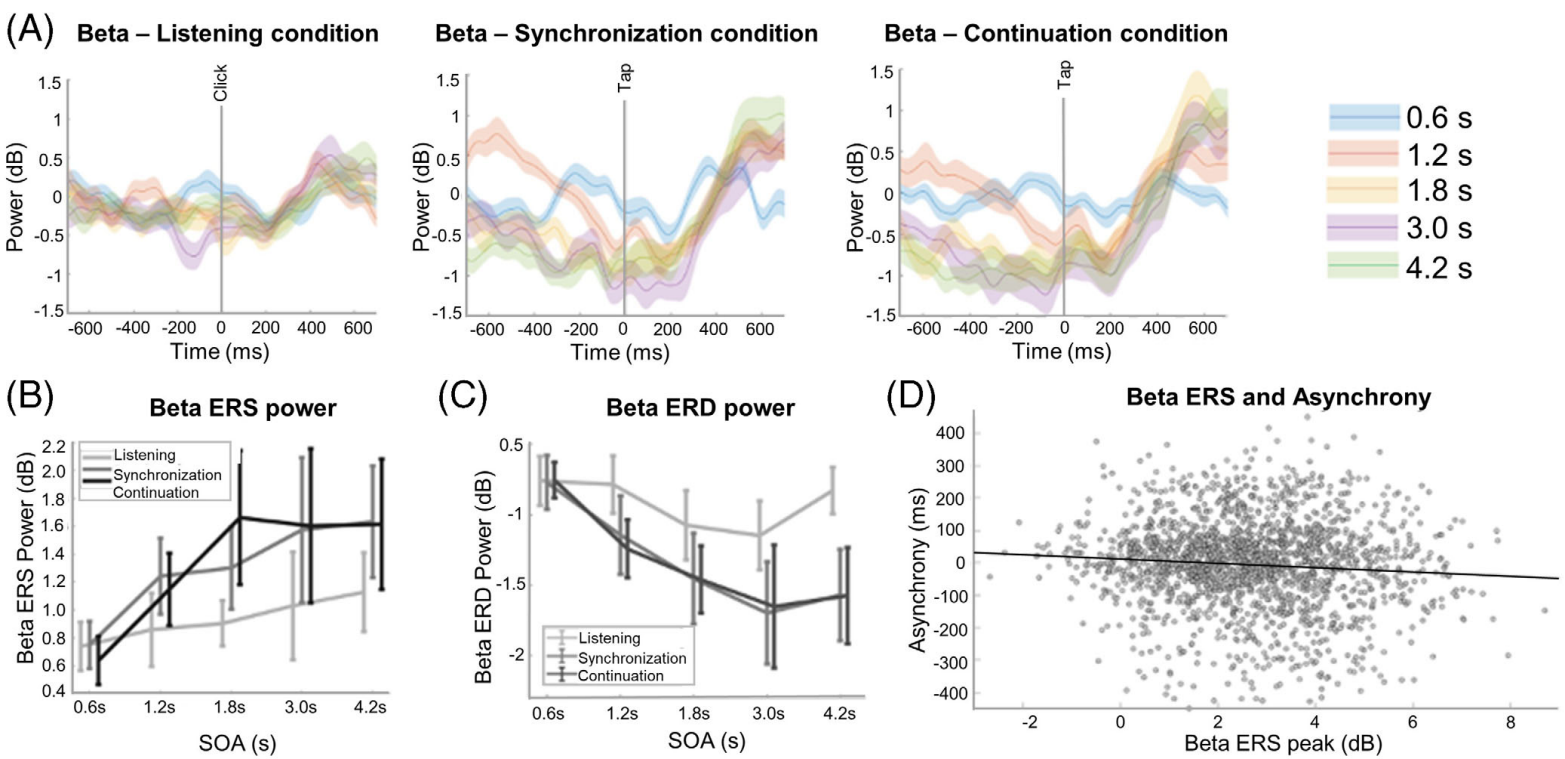
Stimulus‐onset asynchrony (SOA) and task effects on motor beta oscillation. (A) The motor beta oscillation under different SOA conditions during three distinct tasks. (B) Beta event‐related desynchronization (ERD) increased when SOA lengthened for three tasks. (C) Beta event‐related synchronization (ERS) increased when SOA lengthened for three tasks. (D) Beta ERD peak can predict asynchrony. The error bars denote the standard error.

The selected motor sensors were located at the frontal–parietal area and were left dominant (Figure 2B). A typical beta ERD and ERS can be observed in Figure 3A.

For the beta ERD peak, there was a significant main effect of task (*F*(2,20) = 16.061, *p* < .001, *η* ^2^ = 0.616). The ERD peak was smaller in the listening task compared with the synchronization task (*p* = .012) and continuation task (*p* = .007). There was also a significant main effect of SOA (*F*(4,40) = 19.541, *p* < .001, *η* ^2^ = 0.661). The ERD peak magnitude was smaller when SOA was 0.6 s compared to 1.2 s (*p* = .031), 1.8 s (*p* = .001), 2.4 s (*p* = .008), and 3.0 s (*p* = .004). See Figure 3B for more details.

For the beta ERS peak, there was a significant main effect of task (*F*(2,20) = 10.204, *p* < .001, *η* ^2^ = 0.505). The ERS peak was smaller in the listening task compared with the synchronization task (*p* = .028) and continuation task (*p* = .007). In addition, there was a significant main effect of SOA (*F*(4,40) = 13.179, *p* < .001, *η* ^2^ = 0.569). The ERS peak was smaller when SOA was 0.6 s compared to 1.2 s (*p* = .002), 1.8 s (*p* = .001), 2.4 s (*p* = .024), and 3.0 s (*p* = .001). The ERS peak was smaller when SOA was 1.2 s compared to 4.1 s (*p* = .032). See Figure 3C for more details.

**FIGURE 4:**
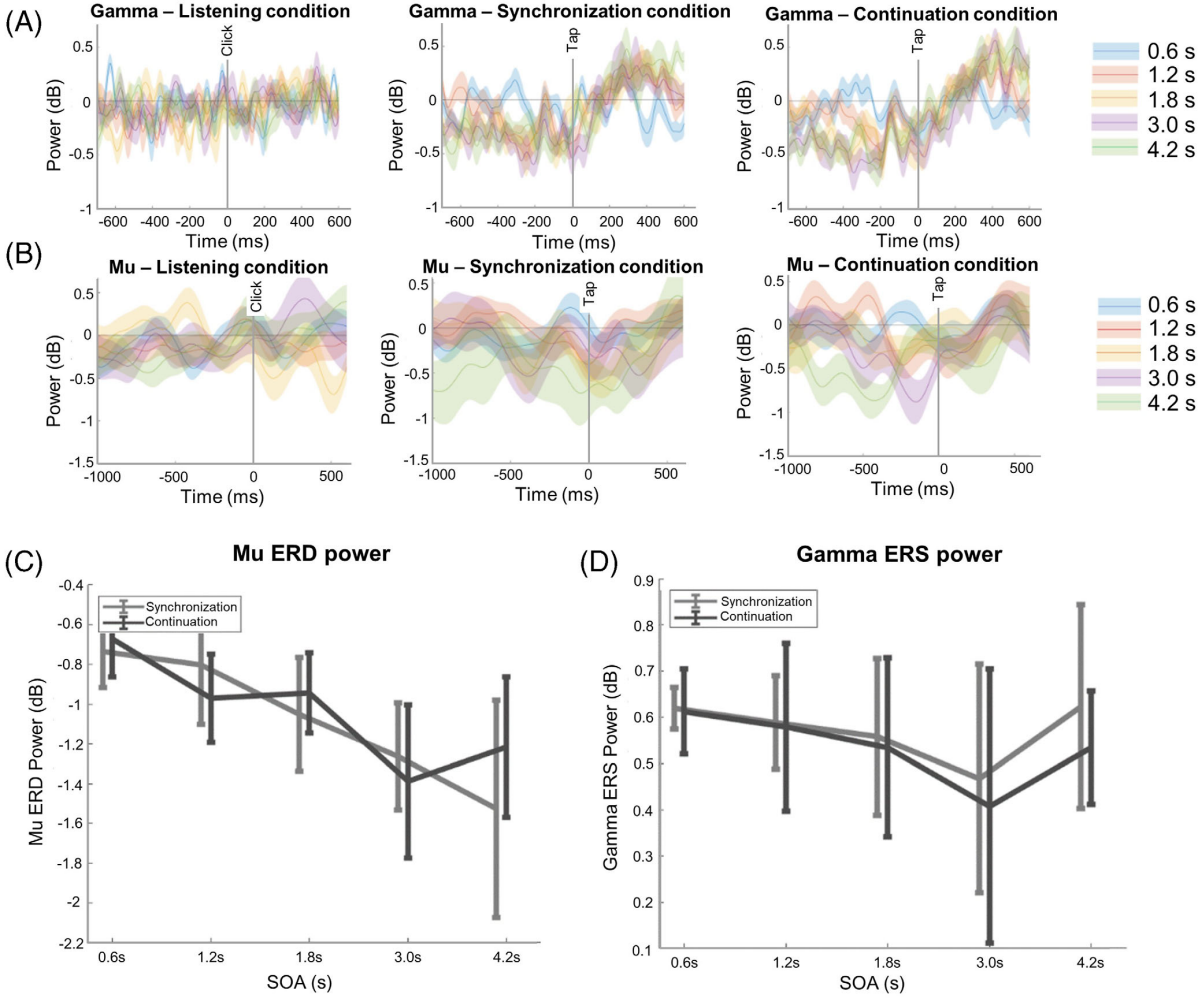
Stimulus‐onset asynchrony (SOA) and task effects on motor mu and gamma oscillation. (A) The motor gamma oscillation under different SOA conditions during three distinct tasks. (B) The motor mu oscillation under different SOA conditions during three distinct tasks. (C) Mu event‐related desynchronization (ERD) peak was significantly affected by SOA. (D) Gamma event‐related synchronization (ERS) peak was not significantly affected by SOA or task. The error bars denote the standard error.

Activities of mu oscillation desynchronized more with long SOAs during tasks with overt movements. Specifically, in the listening task, we did not find significant changes in mu activities (Figure 4B); However, in the synchronization and continuation tasks, the mu ERD peaks decreased with SOA (*F*(4,40) = 5.875, *p* < .001, *η* ^2^ = .370; Figure 4C).

For activities of gamma oscillation, in the listening task, we did not find significant changes in gamma activities (Figure 4A), and the gamma ERS peaks showed no significant changes with SOA and conditions (Figure 4D).

### Beta synchronization ERS predicted behavioral asynchronies

Based on the above results, the mu ERD, beta ERD, and ERS peaks changed with SOA in the synchronization task. We then adopted these three neuronal activities for the linear mixed regression model. The modeling results revealed that the beta ERS peak can significantly predict the asynchrony (beta = −5.590, *F*(1, 1977.2) = 7.4129, *p* = .007; Figure 3D). The greater the beta ERS peak was, the more positive the asynchrony was (Figure 3D).

## DISCUSSION

The current study revealed three main findings. First, beta oscillation in the motor system changes with rhythm tempo with and without overt movements. Second, the beta ERS peak could predict behavioral asynchrony during the synchronization task. Third, in the synchronization task, mu oscillation changes with rhythm tempo while the gamma oscillation does not.

Consistent with previous reports, we observed that the beta ERS and ERD increase as SOA gets longer (e.g., Seeber et al., 2016; Toma et al., 2002). Moreover, the modulation of SOA on beta activity can be found even during passive listening. This suggests that beta oscillations possibly are related to two functions: not only movement inhibition but also temporal prediction.

By acting as an active motor inhibitor in the condition of short SOA, the desynchronized beta oscillations may be sustained to maintain fast and continuous movements. The observation of smaller ERS and ERD peaks may indicate that synchronization with short SOA requires smaller cortical populations (Buzsaki, 2006). It is then plausible to assume that fast movements are easier to maintain and may require fewer cognitive resources (Park et al., 2017; van der Wel et al., 2009).

As a part of the temporal prediction system, beta oscillations may be involved in actively predicting the temporal aspect of movement by varying the ERS and ERS peak magnitudes. Since the tempo of the passive listening task was the same as that of the synchronization task, subjects may have actively engaged their imaginary motor in their mind, resulting in changes in beta activity. The beta activity could be the signal of a simulated motor efference to sensory cortices for encoding timing information (Patel & Iversen, 2014). During passive listening, the change of beta oscillation with SOA might reflect the simulation process.

A negative correlation was observed between the beta ERS and behavioral asynchrony in the synchronization task. Similarly, using a time reproduction task, researchers found that the amplitude of beta ERS could predict the length of the reproduced duration (Kononowicz et al., 2015). Aligned with such observations, with an SOA ranging from 300 to 1200 ms, Fujioka et al. (2012) reported that the ERS amplitude of beta oscillation over auditory cortices and motor‐related areas correlated with the tempo information. Based on the above experimental results, it is reasonable to believe that the beta ERS represents a timing information integration process, which reflects subjective timing information (Tan et al., 2014; Torrecillos et al., 2015). In our study, the negative correlation may also indicate the subjective time differences between predictive and reactive taps. Predictive taps occur when the subjective elapsed time of participants reaches the SOA, especially when the SOA is shorter than 3 s. Alternatively, when the SOA is longer, reactive taps occur when subjects react to the click onset before the subjective elapsed time reaches the SOA. We speculate that the difference in beta ERS magnitude results from different perceptual feedback and the timing integration processes after the two types of taps. Thus, beta oscillation could be a reliable neural indicator to distinguish between predictive tapping with short SOA and reactive tapping with long SOA. It is important to note that beta oscillation may also contribute to the temporal organization in motor control because it varies with short or long SOA. The border line here between short and long is around 3 s, which represents a distinct logistic temporal frame in human cognition verified by evidence from various fields (Zhao et al., 2022).

Additionally, we found that the SOA affected mu oscillations during sensorimotor synchronization; however, there was no significant change in mu oscillations during the listening task, where no movement was required. Based on this result, mu oscillations may be related only to changes in motor components, such as changes in motor speed; mu oscillations are likely not involved in the temporal processing of rhythmic auditory stimuli either. On the other hand, gamma oscillations are unaffected by the SOA. These results are consistent with previous studies that have shown gamma oscillations to be more related to local information processing (Muthukumaraswamy, 2010).

## CONCLUSIONS

The present study supports the active functional role of beta oscillations in the motor cortex during temporal anticipation, particularly the beta ERS. Additionally, mu oscillations can reflect movement frequency, whereas gamma oscillations may involve a bottom‐up process. Combined, these motor‐related oscillations facilitate sensorimotor synchronization with different functions.

## CONFLICT OF INTEREST STATEMENT

The authors declare that there are no conflicts of interest.

## ETHICS STATEMENT

The study was approved by the ethical board of Ludwig Maximilian University of Munich.
